# Michigan’s Veterinary Act

**Published:** 1899-05

**Authors:** 


### EDITORIAL.

### MICHIGAN'S VETERINARY ACT.

Michigan has established by legislative enactment a State Veterinary Board. The act established as a standard of requirements a diploma from schools having a curriculum of two years of six months each. This concession to schools with so short a term is a severe blow to the efforts being put forth at Detroit, where a three-term school exists, and concedes to the Grand Rapids school nothing of vantage when we understand that this school pledged itself to extend its curriculum if the requirements of the American Veterinary Medical Association were adopted. The third section of the act, referring to those entitled to its provisions, states "not aliens," which, while obscure, would indicate that it is only intended for citizens of the State. Non-graduates are eligible for examination. The subjects demanded are anatomy, physiology, veterinary materia medica, and pathology, and an average of not less than 75 per cent. upon all questions is to be obtained. Section twelve says: "Nothing in this Act shall prohibit any person from treating any domestic animal, and only applies to the use of the professional title and degrees, or their abbreviations thereof."

This bill may prove of value as an entering wedge for future amendment; but the low standard recognized, the subjects designated as required for instruction, the open door for violation of its provisions, the absence of suitable provision for visiting veterinarians and those living on the border-lines of adjacent States, leaves much to be desired in the way of higher veterinary education, and vouchsafes but little protection for the people and their livestock interests from preying charlatans and dangerous practitioners as having only the authority of a diploma obtained from schools whose easy requirements demand only a meagre education in exchange for a sheepskin.

